# Synergistic Integration of Recombinase Polymerase Amplification and CRISPR–Cas12a Enables High‐Sensitivity Detection of *Mycoplasma bovis*

**DOI:** 10.1002/mbo3.70435

**Published:** 2026-10-12

**Authors:** Diba Yaghoubi, Viju V. Pillai

**Affiliations:** ^1^ Department of Comparative Pathobiology College of Veterinary Medicine Purdue University West Lafayette Indiana USA; ^2^ Wille M. Reed Animal Disease Diagnostic Laboratory Purdue University West Lafayette Indiana USA

**Keywords:** bison, CRISPR‐Cas12a, mycoplasma bovis, on‐site detection, recombinase polymerase amplification

## Abstract

*Mycoplasma bovis* is a major pathogen of cattle, causing substantial economic losses in the global cattle industry and emerging as a serious threat to North American bison (*Bison bison*). Effective outbreak management is hindered by the lack of rapid diagnostic methodologies deployable in field settings. Current testing approaches are limited by slow turnaround times for microbial culture and the dependence of qPCR on centralized laboratory infrastructure, both of which are poorly suited for field settings. To address this gap, we developed a highly sensitive, isothermal diagnostic platform coupling Recombinase Polymerase Amplification with CRISPR‐Cas12a detection targeting the conserved uvrC gene. The optimized assay requires less than 1 h at 37°–39°C and achieves an analytical limit of detection of approximately 8 copies, comparable to that of the gold‐standard quantitative PCR. Clinical validation using 110 samples from bison and cattle demonstrated 100% concordance with PCR and complete differentiation from closely related species, including *Mycoplasma canis* and *Mycoplasma felis*. Overall, these data establish the RPA–CRISPR/Cas12a assay as a sensitive and specific isothermal approach for detecting *M. bovis* in clinical specimens, providing a field‐adaptable framework for future instrument‐free surveillance in resource‐limited agricultural settings and supporting improved herd health management in both commercial cattle and bison conservation operations.

## Graphical abstract

This graphical abstract illustrates a rapid diagnostic platform for *Mycoplasma bovis* detection using Recombinase Polymerase Amplification coupled with CRISPR–Cas12a targeting the uvrC gene. The assay operates at 37°C–39°C, delivers results within 1 h, and demonstrates complete concordance with PCR in cattle and bison clinical samples.

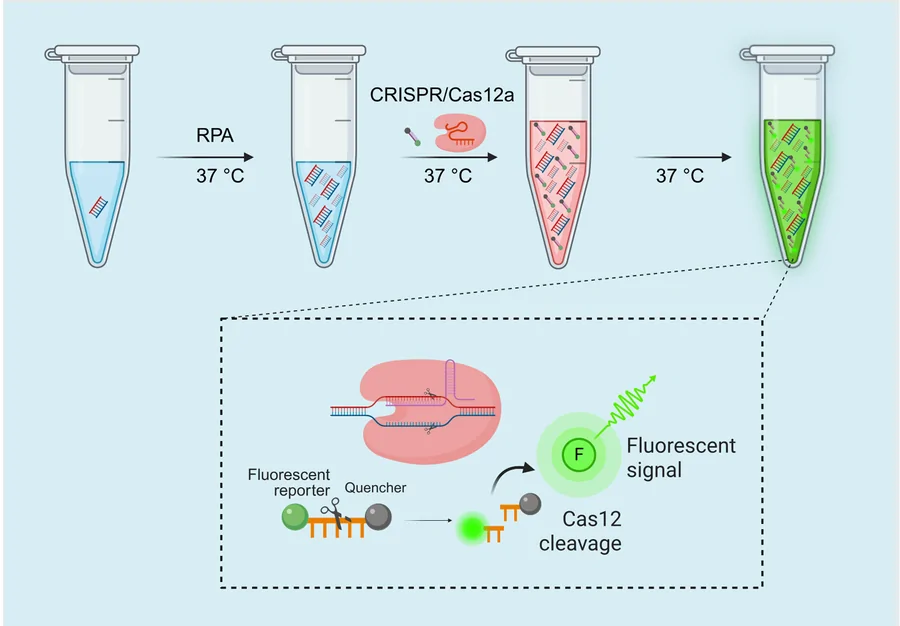

## 1 Introduction

*Mycoplasma bovis* is a major multi‐systemic pathogen of Bovidae that causes substantial economic losses in the global cattle industry and is an emerging health threat to North American bison (*Bison bison*) herds (Caswell et al. 2010; Martin et al. 2025; Su et al. 2025). In cattle, *M. bovis* is a primary causative agent of chronic pneumonia, polyarthritis, and otitis media, especially in feedlot animals and calves (Caswell et al. 2010; Maunsell et al. 2011; Maunsell and Donovan 2009). It is also an important cause of mastitis in dairy herds (Maunsell et al. 2011). The organism often evades host immune defenses through variable surface lipoprotein expression (Bürki et al. 2015). In bison, the pathogen is particularly virulent, with outbreaks frequently associated with high morbidity and mortality rates, often manifesting as severe, necrotizing pneumonia resistant to conventional antimicrobial therapy (Buttke et al. 2025; Janardhan et al. 2010). Control of *M. bovis* is further complicated by intrinsic resistance to β‐lactam antimicrobials and the limited efficacy of available commercial vaccines, making early detection and segregation of infected animals central to disease management (Buttke et al. 2026).

Effective control in both cattle and bison operations is constrained by the limitations of existing diagnostic modalities. Traditional microbiological culture remains definitive but is labor‐intensive and may require days to weeks because of the fastidious growth requirements of the organism, a turnaround time that is incompatible with outbreak decision‐making (Buttke et al. 2026). Serologic assays provide faster results but often cannot reliably distinguish active infection from past exposure or maternal antibody (Register et al. 2013). Consequently, nucleic acid amplification tests, such as quantitative polymerase chain reaction (qPCR), have become the gold standard due to their high analytical sensitivity and specificity (Parker et al. 2018). However, PCR‐based methods are tethered to centralized laboratories as they typically require centralized laboratory infrastructure and trained personnel, creating logistical bottlenecks. This limitation is especially problematic for the bison industry, where repeated animal handling for resampling is stressful and can pose substantial safety risks, underscoring the need for a rapid “chute‐side” diagnostic that can deliver actionable results during a single handling event.

To address this gap, isothermal amplification techniques such as recombinase polymerase amplification (RPA) have proven to be attractive alternatives (Tan et al. 2022). Unlike PCR, RPA operates at a constant low temperature (37°C–42°C) and can tolerate inhibitors commonly present in clinical matrices, including nasal swabs and lung tissue‐derived extracts (Lobato and O'Sullivan 2018). However, standalone isothermal assays often suffer from non‐specific amplification, leading to false‐positive results when coupled with non‐specific detection methods like intercalating dyes (Daher et al. 2016; Lobato and O'Sullivan 2018; Tan et al. 2022). To resolve this, integrating CRISPR‐based detection can provide an additional layer of sequence specificity (Kaminski et al. 2021). The Type V effector Cas12a (Cpf1) possesses a unique collateral trans‐cleavage activity. Upon binding a target DNA sequence complementary to its guide RNA (crRNA), Cas12a is activated and non‐specifically cleaves nearby single‐stranded DNA (ssDNA) reporters (Chen et al. 2018), generating a fluorescence signal.

In this study, we developed and validated a rapid, highly sensitive diagnostic assay for *M. bovis* by coupling RPA pre‐amplification with CRISPR‐Cas12a detection. The assay targets uvrC, a conserved housekeeping gene that has been widely used as a molecular marker for the identification of *M. bovis*. (Subramaniam et al. 1998; Thomas 2004). Although uvrC is broadly conserved among Mycoplasma species, species‐specific sequence polymorphisms within the selected target region permit differentiation of *M. bovis* from closely related species. The gene is also highly conserved among *M. bovis* isolates and is a preferred species‐specific qPCR target for routine diagnostic detection in cattle (Clothier et al. 2010). We evaluated multiple guide RNA configurations to optimize detection performance in clinical samples from both bison and cattle. By combining the amplification efficiency of RPA with the sequence specificity of CRISPR‐Cas12a, this platform aims to provide a sensitive and field‐adaptable approach to support surveillance and control of *M. bovis* infection in bovine populations.

## 2 Materials and Methods

### 2.1 Sample Collection and DNA Extraction

Clinical samples (lung tissue, nasopharyngeal swabs and milk) were obtained from the Indiana Animal Disease Diagnostic Laboratory (ADDL), West Lafayette, IN, USA and were submitted part of routine testing and screening. Genomic DNA (gDNA) was extracted from clinical specimens or diagnostic sample lysates using the Invitrogen Genomic DNA Mini Kit (Thermo Fisher Scientific) according to the manufacturer's instructions. DNA concentration and purity were assessed using a NanoDrop spectrophotometer (Thermo Scientific). The same purified DNA extracts used for reference PCR/qPCR testing were used for RPA‐CRISPR/Cas12a testing, allowing direct comparison of assay performance on identical templates.

### 2.2 PCR Amplification of the *M. bovis* uvrC Gene

*M. bovis* gDNA was extracted from lung tissue from a PCR‐confirmed *M. bovis*–infected bison with gross pulmonary lesions. The uvrC gene in *M. bovis* gDNA was amplified using PCR on a thermal cycler (ThermoFisher Scientific) using primers flanked with restriction sites, R1‐RE and R2‐RE (Table 1). Primers targeted a 558 bp fragment of the uvrC gene (nucleotide positions 1140–1697; GenBank accession no. AF003959.1). Sequences were designed using NCBI BLAST and synthesized by Invitrogen (Table 1). Each 50 µL single‐tube reaction contained 10 µL of Q5 Reaction Buffer, 2.5 µL of forward primer (10 µM), 2.5 µL of reverse primer (10 µM), 1 µL of *M. bovis* genomic DNA, 1 µL of deoxynucleoside triphosphates (dNTPs), 0.5 µL of Q5 High‐Fidelity DNA Polymerase (New England Biolabs, USA), and 32.5 µL of nuclease‐free water (ThermoFisher Scientific). PCR cycling conditions were as follows: initial denaturation at 98°C for 30 s; 25–35 cycles of denaturation at 98°C for 10 s, annealing at 60°C for 30 s, and extension at 72°C for 45 s; final extension at 72°C for 2 min; and a hold at 10°C. Amplicons were purified using the Zymo DNA Clean & Concentrator−5 kit (Zymo Research). Purified DNA was digested with SpeI‐HF and EcoRV‐HF restriction enzymes (New England Biolabs) and resolved on a 1.5% agarose gel. The target band was excised and purified using the Monarch DNA Gel Extraction Kit (New England Biolabs).

**Table 1:** Primers used for PCR and RPA reaction.

RPA primers	Sequence
PP1	F: CTTAAAACGCTTAATATAAACATCCCTGT R: AACTTGAATTTGAACTAAGTAGTTGTATAG
PP2	F: AACGCTTAATATAAACATCCCTGTTATTG R: AACTTGAATTTGAACTAAGTAGTTGTATAG
PP3	F: GTAGCAACAAAAACACTAAAGATTATGACT R: AACTTGAATTTGAACTAAGTAGTTGTATAG
PP4	F: CTTAAAACGCTTAATATAAACATCCCTGT R: TCCTTTTATGTTTCTTAGTTTGCCTTCTAGTG
PP5	F: AACGCTTAATATAAACATCCCTGTTATTG R: TCCTTTTATGTTTCTTAGTTTGCCTTCTAGTG
PP6	F: TAAATGAGCGCAGTGCTGATGTTGAAT R: AACTTGAATTTGAACTAAGTAGTTGTATAG
PP7	F: GTAGCAACAAAAACACTAAAGATTATGACT R: TCCTTTTATGTTTCTTAGTTTGCCTTCTAGTG
PP8	F: TAAATGAGCGCAGTGCTGATGTTGAAT R: TCCTTTTATGTTTCTTAGTTTGCCTTCTAGTG
R1‐RE	GAGAACTAGTCCTGTCGGAGTTGCAATTGTT
R2‐RE	AATGCTTTTGGCAATATTAAGTGGA

### 2.3 Generation of Standard Plasmid Control (P‐uvrC)

The purified and gel extracted 558 base pair fragment of the uvrC gene was cloned into the pCR‐Blunt II‐TOPO vector (Zero Blunt TOPO PCR Cloning Kit, Invitrogen), which had been pre‐digested with SpeI‐HF and EcoRV‐HF restriction enzymes (New England Biolabs). The PCR primers used for amplification were also designed to include SpeI‐HF and EcoRV restriction sites at their 5′ ends, enabling directional cloning and preventing inverted ligation. Following ligation, the construct was transformed into One Shot Stbl3 Chemically Competent *E. coli* (Thermo Scientific). Transformants were selected on kanamycin‐impregnated LB agar plates. Plasmid DNA was extracted using the ZymoPURE Plasmid Miniprep Kit (Zymo Research), and the insert was sequence‐confirmed via Sanger sequencing. The plasmid copy number was calculated based on the molecular weight and concentration, and this standard (p‐uvrC) was used for all subsequent sensitivity assays.

### 2.4 RPA Reactions and Primer Design

RPA reactions were performed using the TwistAmp Basic Kit (TwistDX, UK). Primers targeting a 100–400 bp region within the uvrC gene were designed using Primer3 (Table 1) or were reported previously (Zhao et al. 2018). Specificity was validated in silico via NCBI BLAST. Each 50 µL reaction contained: 29.5 µL rehydration buffer, 2.4 µL each of forward and reverse primers (10 µM), template DNA, and nuclease‐free water. The reaction was initiated by the addition of 2.5 µL of 280 mM magnesium acetate. Tubes were incubated at 39°C for 20 min, with a brief vortex and spin‐down performed at the 4‐min mark to ensure homogenous amplification.

### 2.5 CRISPR/Cas12a Detection Reaction and crRNA Design

Cas12a detection was performed using EnGen LbCas12a (New England Biolabs). CRISPR guide RNAs (crRNAs) were designed using CHOPCHOP to target the RPA‐amplified uvrC region. Ribonucleoprotein (RNP) complexes were pre‐assembled by incubating 50 nM LbCas12a and 62.5 nM crRNA for 10 min, after which the remaining reaction components were added. Following RNP formation,10× NEBuffer r2.1 and a ssDNA fluorophore‐quencher reporter (5'‐/56‐FAM/TTATTATT/3BHQ/−3’) were added to a final concentration of 0.5–5.0 µM. A final concentration of 0.75 µM was used for all subsequent clinical validations. Fluorescence kinetics were monitored on a QuantStudio 3 Real‐Time PCR System (Applied Biosystems) at 37°C for 60 min (FAM channel). Endpoint fluorescence was visually assessed using a UV light transilluminator. Fluorescence positivity was defined relative to background‐control reactions. During assay optimization, the no‐template control was used as the negative/background control. For clinical validation, background fluorescence was defined using both the no‐template control and qPCR‐confirmed *M. bovis*–negative clinical samples. Fluorescence was measured over a 60 min period using the real‐time PCR system. Clinical samples were classified as positive when fluorescence intensity increased above background and was clearly distinguishable from negative controls by the 30 min endpoint. Samples with fluorescence signals comparable to background controls throughout the reaction were classified as negative.

### 2.6 Statistical Analysis

Assay optimization experiments were performed with at least three technical replicates unless otherwise indicated, and data are presented as mean ± standard error of the mean (SEM). Clinical validation was performed using independent clinical DNA extracts, and RPA‐CRISPR/Cas12a results were interpreted qualitatively as positive or negative using the fluorescence positivity criteria described above. Diagnostic performance was evaluated at the sample level using reference PCR/qPCR classification as the comparator. True positives (TP), false negatives (FN), false positives (FP), and true negatives (TN) were determined from a 2 × 2 contingency table. Sensitivity and specificity were calculated as TP/(TP + FN) and TN/(TN + FP), respectively. Overall concordance was calculated as (TP + TN)/total number of samples. Because the observed point estimates were at the boundary of the parameter space, with values of 100%, exact two‐sided 95% confidence intervals were calculated using the Clopper–Pearson method, which is appropriate for binomial proportions at the boundary of the parameter space. The relationship between reference qPCR Ct values and RPA‐CRISPR/Cas12a fluorescence intensity was assessed using Spearman correlation analysis. Data analysis and visualization were performed using GraphPad Prism (v10.6.1) and RStudio (v2024.04). Correlation plots and heatmaps were generated using the *ggplot2* package in R.

## 3 Results

### 3.1 Optimization of RPA Primers Targeting the *M. bovis* uvrC Gene

A 558‐bp region of the uvrC gene that was PCR‐amplified from *M. bovis* genomic DNA and cloned into a plasmid vector (p‐uvrC), served as the standard DNA control for the reactions performed (Figure 1A). Eight primer‐pair combinations (PP1–PP8) targeting uvrC and designed to amplify ~100–400 bp fragments were evaluated in RPA reactions at 39°C for 20 min. Expected amplicon sizes ranged from approximately 100–400 bp, and most primer pairs yielded distinct bands at the expected sizes (Figure 1B). Of the eight primer pairs tested, PP3–PP8 produced strong, specific bands using the p‐uvrC standard. Among the best‐performing primer pairs, PP7 was selected for all subsequent RPA–CRISPR/Cas12a optimization and validation experiments because it demonstrated robust and specific amplification and provided suitable target sites for the design of three distinct gRNAs (gRNA1–gRNA3) within the amplified region. In addition, RPA performed using DNA extracted from bison lung tissue confirmed to have *M. bovis* infection by histopathology, and PCR also generated the target amplicon (Figure 1B). Although minor non‐specific bands were observed in some gDNA reactions, the primary target was distinct and sufficient for downstream detection.

**Figure 1:**
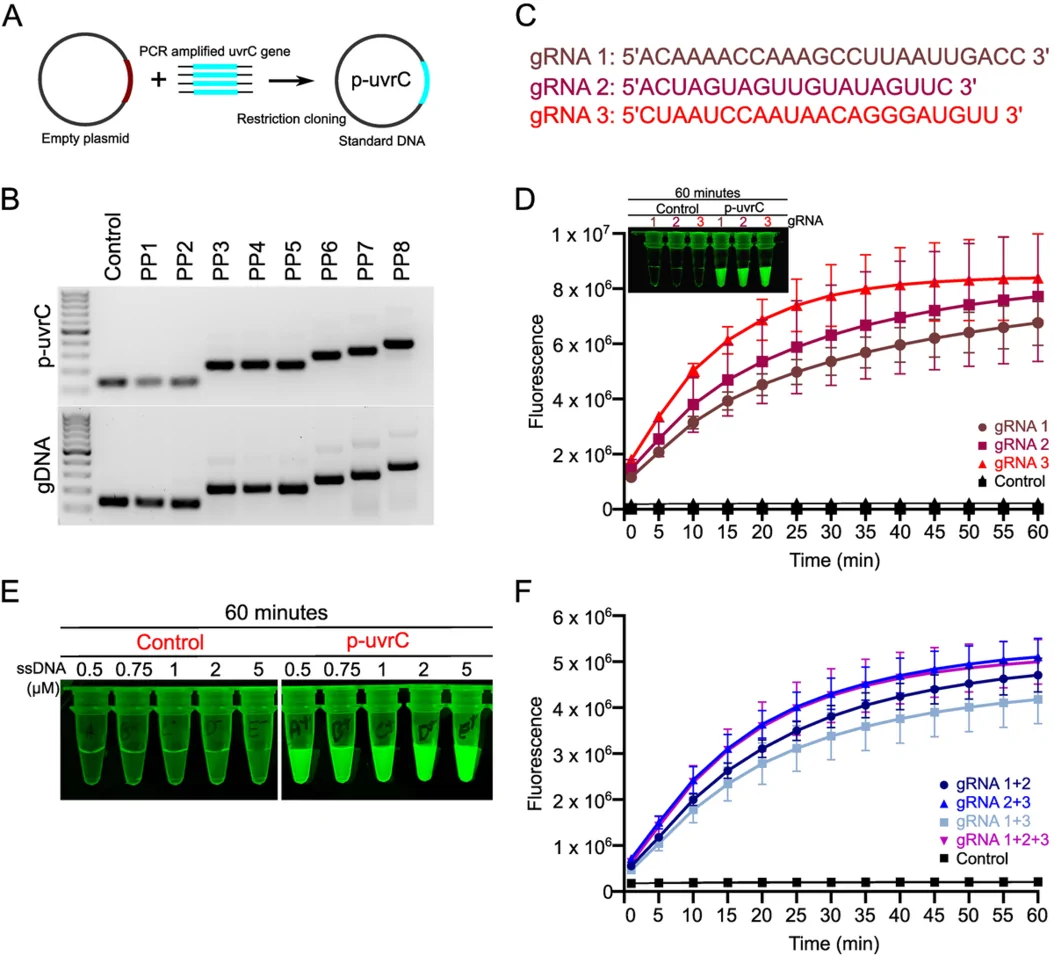
Optimization of RPA primers and CRISPR/Cas12a detection for the *Mycoplasma bovis* uvrC gene. (A) Schematic of p‐uvrC generation by restriction cloning of a 558‐bp uvrC fragment, which was used as the standard DNA template. (B) Screening of eight RPA primer pairs (PP1–PP8) targeting uvrC; PP3–PP8 produced strong, specific amplicons, and target amplification was also detected in DNA from *M. bovis*‐positive bison lung. (C) Sequence of three CRISPR guide RNAs (gRNA1–gRNA3) designed within the amplified uvrC region adjacent to 5′‐TTTG‐3′ PAM sites. (D) Comparison of Cas12a fluorescence kinetics showed that all three guides detected uvrC, with gRNA3 producing the fastest and strongest signal. (E) Optimization of ssDNA‐FQ reporter concentration identified 0.75 μM as the best condition for visual detection with minimal background, although maximal fluorescence was reached at 5 μM. (F) All guide RNA combinations produced detectable fluorescence with similar kinetics, although gRNA2 + 3 and gRNA1 + 2 + 3 showed slightly faster onset.

### 3.2 Optimized Cas12a Trans‐Cleavage Kinetics Enable Rapid Target Detection

Three CRISPR guide RNAs (gRNA1, gRNA2, and gRNA3) were designed to target distinct sites within the amplified *M. bovis* uvrC region, each adjacent to a 5′‐TTTG‐3′ protospacer‐adjacent motif (PAM) and selected to have low sequence homology to other Mycoplasma species (Figure 1C). Each guide was evaluated in a CRISPR/Cas12a fluorescence reporter assay using p‐uvrC standard DNA as template. All three guides enabled Cas12‐mediated detection of uvrC, producing a green fluorescence signal within 30–60 min, whereas a control plasmid lacking the uvrC insert showed no fluorescence. Fluorescence kinetics and signal intensity differed among guides, with gRNA3 producing the fastest and most intense response, followed by gRNA 1 and then gRNA 2 (Figure 1D). Fluorescence for all guides peaked and plateaued by 60 min. The ssDNA‐FQ reporter concentration was then optimized to balance time‐to‐threshold, signal intensity, and background fluorescence. Increasing reporter concentration shortened the threshold time and increased fluorescence intensity; saturated fluorescence values were achieved at 5 μM after 40 min of incubation (Figure 1E). However, higher reporter concentrations were also associated with increased background fluorescence over time in negative controls, and 0.75 μM ssDNA‐FQ provided the best visual readout with minimal background (Figure 1E). Cas12a protein titration indicated best fluorescence intensity at 50 nM (data not shown). Using the optimized CRISPR/Cas12a conditions, guide combinations (gRNA 1 + 2, gRNA 2 + 3, gRNA 1 + 3, and gRNA 1 + 2 + 3) were then tested in the same CRISPR/Cas12a fluorescence reporter assay. All combinations generated readily detectable fluorescence with broadly similar kinetics, although overall intensity followed a lower trend than the single‐guide reactions. gRNA 2 + 3 and gRNA 1 + 2 + 3 combinations showed a slightly faster onset of fluorescence compared to the other combinations (Figure 1F).

### 3.3 RPA Pre‐Amplification Is Essential for Cas12a‐Mediated Detection of *M. bovis* Genomic Dna

When *M. bovis* genomic DNA from three PCR‐confirmed positive samples was tested directly in the CRISPR/Cas12a reaction, no fluorescence signal was detected with any of the guide RNAs, whereas the control p‐uvrC standard produced bright fluorescence (Figure 2A) with all three gRNAs. A plasmid with no uvrC sequence served as the negative control and was negative as expected. However, when the same genomic DNA samples were first subjected to RPA amplification and the resulting amplicons were used as input for CRISPR/Cas12a detection, positive fluorescence signals were obtained. Under these conditions, all gRNAs and gRNA combinations generated comparable fluorescence signals, with gRNA 2 or gRNA 3 yielding modestly higher signals, followed by the gRNA 2 + 3 combination and then the gRNA 1 + 2 + 3 combination (Figure 2B,C). Fluorescence increased over time, peaked at approximately 20–25 min, and then plateaued for all guides and combinations tested (Figure 2D). Based on these findings, subsequent experiments were performed using the gRNA 2 + 3 combination because it generated fluorescence signals comparable or modestly higher than those of the three‐guide mixture while reducing assay complexity. In addition, requiring recognition by two guides provided a practical balance between signal strength and assay stringency relative to a single‐guide reaction.

**Figure 2:**
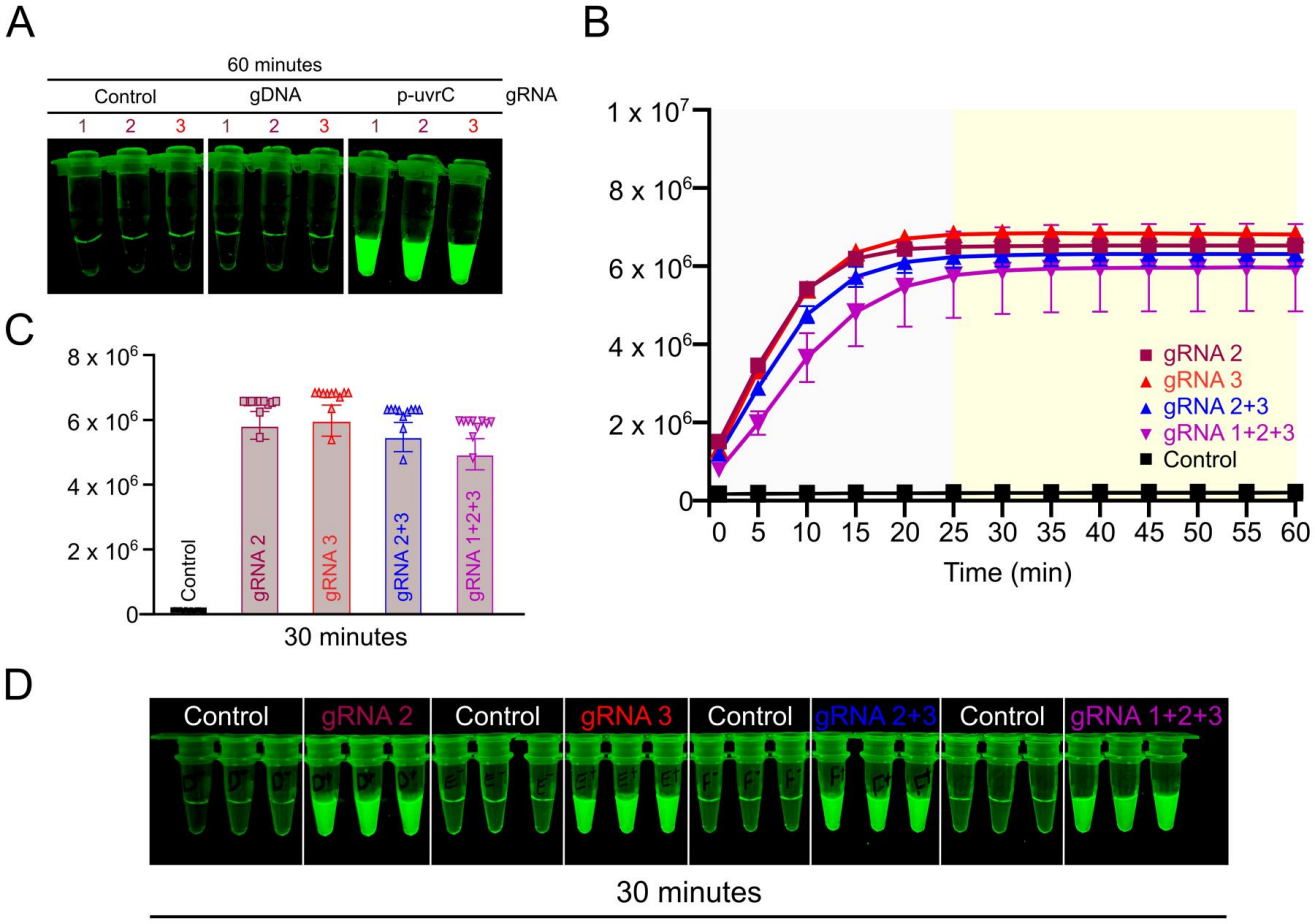
RPA pre‐amplification is required for CRISPR/Cas12a detection of Mycoplasma bovis genomic DNA. (A) Direct testing of genomic DNA from three PCR‐confirmed *M. bovis*‐positive samples in the CRISPR/Cas12a assay produced no detectable fluorescence, whereas the p‐uvrC control was strongly positive. (B, C) After RPA pre‐amplification, the same samples generated positive fluorescence signals, with the strongest responses observed for gRNA2, gRNA3, gRNA2 + 3, and gRNA1 + 2 + 3, although not statistically significant. (D) Fluorescence peaked at approximately 30 min and then plateaued.

### 3.4 qPCR Benchmarking Highlights Rpa‐Dependent Cas12 Sensitivity

Quantitative PCR was performed on serial dilutions of the p‐uvrC plasmid to establish a reference relationship between template copy number and amplification signal. Ct values decreased proportionally with increasing plasmid copy number, and qPCR reliably detected as few as ~8 copies, with a Ct of approximately 35, while the highest input tested (8 × 10^7^ copies) was detected at a Ct of approximately 13 (Figure 3A). In contrast, when the same dilution series was tested directly in the CRISPR/Cas12a fluorescence reporter assay (without pre‐amplification), no measurable fluorescence was observed at any copy number, indicating that the analytical sensitivity of Cas12a detection alone was insufficient across the tested range of p‐uvrC inputs (Figure 3B). However, when each dilution was first subjected to RPA pre‐amplification and the resulting products were used as input for CRISPR/Cas12a detection, robust fluorescence signals were obtained across the dilution series, including at the ~8‐copy level, demonstrating that RPA pre‐amplification is required to achieve low‐copy detection in this workflow (Figure 3C ). To express the Cas12a‐only detection threshold in mass units, p‐uvrC plasmid was additionally serially diluted and tested directly in the CRISPR/Cas12a assay without RPA; visible fluorescence was observed only between 700 (1.6 × 10^11^ copies) and 7 ng (1.6 × 10^9^ copies), and no fluorescence was detected, even after 60 min, at concentrations below 7 ng (Figure 3D).

**Figure 3:**
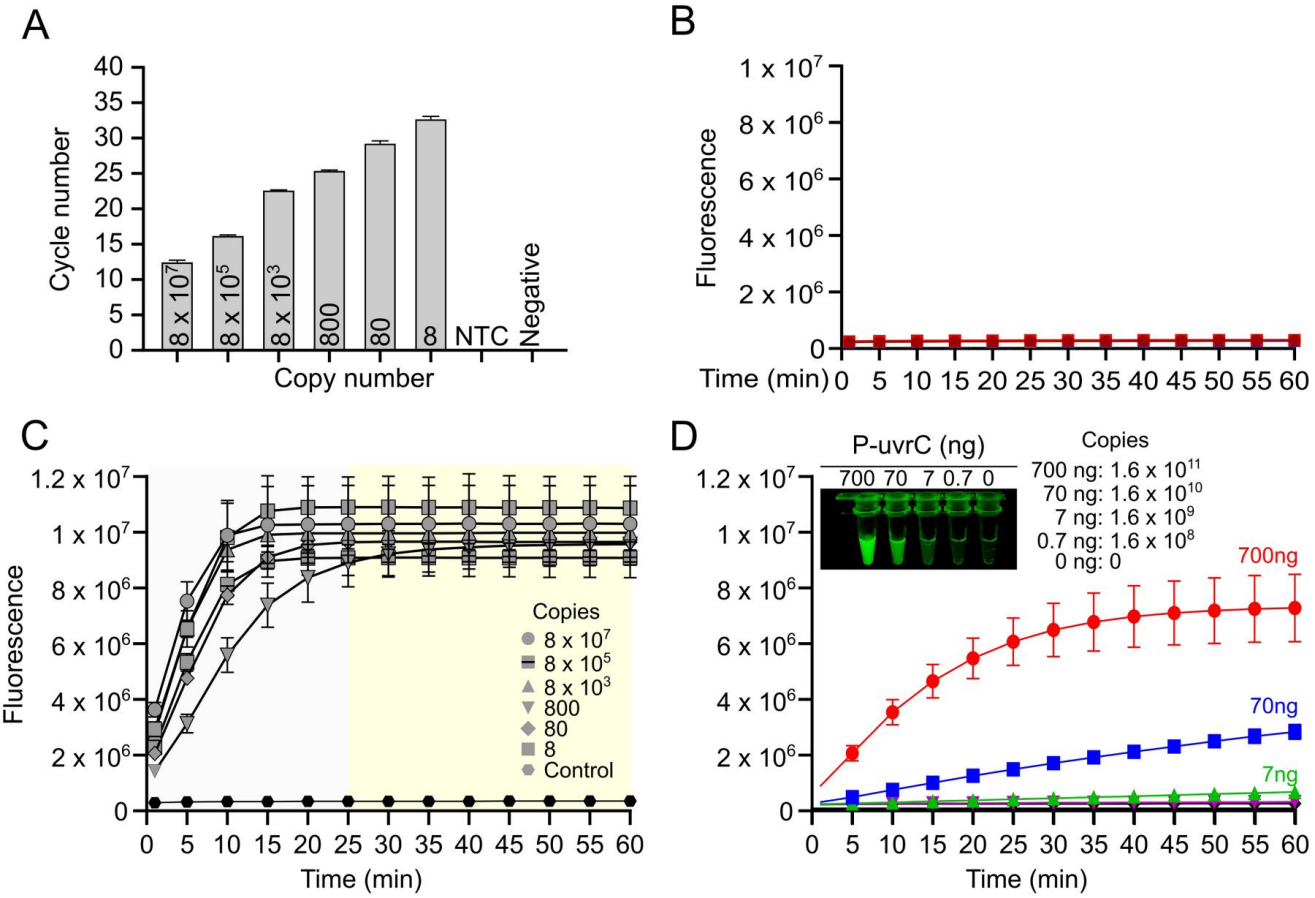
qPCR benchmarking demonstrates the requirement for RPA pre‐amplification to achieve sensitive CRISPR/Cas12a detection. (A) qPCR of serially diluted p‐uvrC plasmid showed decreasing Ct values with increasing template copy number and detected as few as ~8 copies. (B) Direct CRISPR/Cas12a testing of the same plasmid dilution series without pre‐amplification produced no measurable fluorescence. (C) Following RPA pre‐amplification, robust CRISPR/Cas12a fluorescence was observed across the dilution series, including at ~8 copies. (D) Direct CRISPR/Cas12a testing of serially diluted p‐uvrC in mass units showed detectable fluorescence only between 700 (1.6 × 10^11^ copies) and 7 ng (1.6 × 10^9^ copies), with no signal below 7 ng after 60 min. NTC = no template control.

### 3.5 Clinical Validation Demonstrates 100% Concordance With PCR and High Species Specificity of the RPA‐CRISPR/Cas12 Assay

Following analytical optimization, the RPA–CRISPR/Cas12a assay was evaluated using DNA extracted from clinical samples from bison and cattle with PCR‐confirmed Mycoplasma bovis infection, as well as samples positive by PCR for other Mycoplasma species. We also tested M. canis–positive and M. felis–positive samples from dogs and cats. All 50 *M. bovis* PCR‐positive samples were also positive by the RP'A–CRISPR/Cas12a assay (Figure 4A). In contrast, all 13 M. canis PCR‐positive samples and all 10 M. felis PCR‐positive samples were negative with the *M. bovis* RPA–CRISPR/Cas12a assay. Among 37 samples that were PCR‐positive for Mycoplasma spp. (not initially speciated), three were positive by the *M. bovis* RPA–CRISPR/Cas12a assay and 34 were negative; the three RPA–CRISPR/Cas12a–positive samples were subsequently confirmed to be *M. bovis* by species‐specific qPCR, supporting concordance of the assay with PCR‐based identification. Overall, the assay showed 100% sensitivity, 100% specificity, and 100% concordance with reference PCR/qPCR, with exact 95% confidence intervals summarized in Table 2. Ct values of *M. bovis* PCR‐positive samples ranged from approximately 15 to 38, and all samples within this range were detected by the RPA–CRISPR/Cas12a assay following RPA pre‐amplification (Figure 4B). Across *M. bovis*–positive samples, fluorescence intensity showed an inverse association with Ct values (Spearman's ρ = −0.575), indicating that higher fluorescence signals generally corresponded to lower Ct values (Figure 4C).

**Figure 4:**
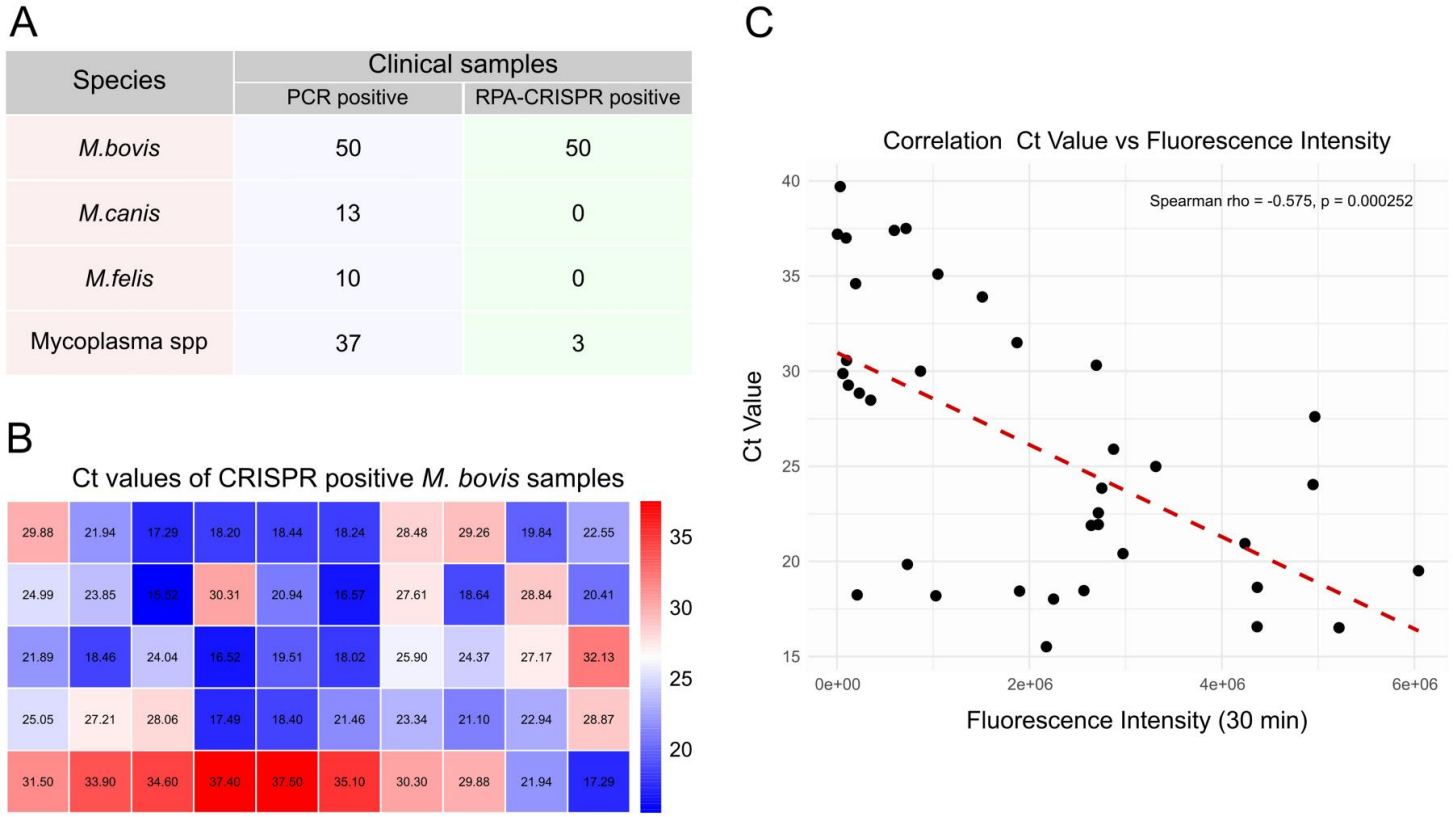
Clinical validation demonstrates complete concordance with PCR and high species specificity of the RPA‐CRISPR/Cas12a assay for Mycoplasma bovis. (A). The assay correctly identified all 50 PCR‐confirmed *M. bovis*‐positive clinical samples and was negative in all M. canis‐positive and M. felis‐positive samples. Among 37 samples positive by PCR for Mycoplasma spp., three were positive by the *M. bovis* RPA‐CRISPR/Cas12a assay and were subsequently confirmed as *M. bovis* by species‐specific qPCR. (B) All *M. bovis*‐positive samples with PCR Ct values ranging from approximately 15–38 were detected by the assay following RPA pre‐amplification. (C) Fluorescence intensity showed an inverse association with Ct value (Spearman's *ρ* = −0.575), indicating that stronger fluorescence generally corresponded to lower Ct values.

**Table 2:** Diagnostic performance of the RPA–CRISPR/Cas12a assay for detection of *M. bovis* in clinical samples (*n* = 110).

Measure	Definition	Result	95% CI
True positives (TP)	Assay positive, *M. bovis* confirmed	53	—
False negatives (FN)	Assay negative, *M. bovis* confirmed	0	—
False positives (FP)	Assay positive, *M. bovis* not confirmed	0	—
True negatives (TN)	Assay negative, *M. bovis* not confirmed	57	—
Sensitivity	TP/(TP + FN)	100% (53/53)	93.3–100%
Specificity	TN/(TN + FP)	100% (57/57)	93.7–100%
Overall concordance	(TP + TN)/total	100% (110/110)	96.7–100%

## 4 Discussion

The development of rapid, highly sensitive point‐of‐care diagnostics is critical for managing *M. bovis* outbreaks and mitigating economic losses associated with bovine respiratory disease. In this study, we successfully established an RPA‐coupled CRISPR‐Cas12a fluorescence assay that matches the low‐copy analytical sensitivity of gold‐standard qPCR while operating under isothermal conditions. Unlike traditional culture methods that require days for confirmation (Parker et al. 2018), or qPCR assays dependent on sophisticated thermal cycling infrastructure, the RPA‐Cas12a platform offers a viable alternative for decentralized testing without compromising analytical rigor.

Several molecular methods have been developed for *M. bovis* detection in bovids, including conventional PCR, qPCR, LAMP, and RPA‐based assays (Higa et al. 2016; Subramaniam et al. 1998; Zhao et al. 2018). PCR and qPCR provide high analytical sensitivity and specificity but require laboratory equipment, trained personnel, and controlled workflows, limiting their utility for chute‐side or field deployment. Loop‐mediated isothermal amplification (LAMP) assays provide an isothermal alternative for molecular detection and can generate rapid visual results with high analytical sensitivity and reduced instrumentation requirements (Higa et al. 2016). However, LAMP often relies on amplification‐based, colorimetric, or turbidity‐based readouts, which may increase the risk of false‐positive interpretation when nonspecific amplification occurs. Additionally, the requirement for multiple primers targeting distinct regions of the same sequence makes LAMP primer design and assay optimization more complicated (Higa et al. 2016). Among other isothermal amplification methods, RPA‐based assays are particularly attractive for field diagnostics because they operate at low constant temperatures without requiring sophisticated instruments. However, RPA alone depends primarily on primer‐mediated target recognition and, when coupled to nonspecific readouts, may be more vulnerable to nonspecific amplification or false‐positive results(Zhao et al. 2018). Thus, the main practical contribution of the present assay is that it combines the speed and low‐temperature requirements of RPA with guide RNA‐ and PAM‐dependent Cas12a recognition, providing a second sequence‐specific detection step after amplification while preserving field‐adaptable assay conditions.

A central finding of this work is the requirement for RPA pre‐amplification to achieve clinically relevant detection limits. Our data revealed that direct Cas12a interrogation of genomic DNA, even with optimized guides, failed to generate detectable fluorescence below 7 ng. This observation aligns with recent characterizations of Cas12a kinetics, which indicate that while the enzyme functions as a powerful signal transducer via trans‐cleavage turnover, it lacks intrinsic amplification capability sufficient for detecting low‐copy targets in complex matrices (Nalefski et al. 2021; Shi et al. 2021; Wang et al. 2025). Importantly, although attomolar detection limits have been reported for purified DNA under highly controlled experimental conditions (Chen et al. 2022; Ghouneimy et al. 2023; Kaminski et al. 2021), such analytical sensitivity may not directly translate to clinical specimens. In infectious disease samples, the extracted nucleic acid typically consists of a heterogeneous mixture of host and pathogen DNA, such that total DNA input overestimates the actual amount of target DNA available for Cas12a recognition. Hence in field conditions, the true pathogen burden might fall below the detection threshold of Cas12a in the absence of pre‐amplification. Incorporation of RPA provides the necessary enrichment of low‐abundance target DNA, whereas Cas12a adds a second layer of sequence‐specific detection by generating signal only when the intended amplicon is present in the appropriate PAM‐dependent context (Chen et al. 2022; Ghouneimy et al. 2023). This design reduces the likelihood that nonspecific amplification products generated during RPA will produce a positive signal, thereby minimizing false positives that commonly affect isothermal amplification methods relying on nonspecific detection chemistries such as intercalating dyes (Lobato and O'Sullivan 2018; Tan et al. 2022).

The optimization of the CRISPR effector step further highlighted the complex kinetics of multiplexed guide RNA strategies. Our optimization of the CRISPR effector step demonstrated that a dual‐guide approach (gRNA 2 + 3) offered a superior balance of performance compared to single or triple‐guide strategies. Interestingly, while some reports suggest that multiplexing guides leads to additive signal intensity (Morales‐Moreno et al. 2023; Zeng et al. 2022), we observed a lower peak fluorescence but a faster time‐to‐threshold with the dual‐guide complex. This likely reflects a kinetic trade‐off; while multiple guides increase the probability of rapid initial target capture (reducing the lag phase), they may compete for the available Cas12a pool, slightly dampening the maximum trans‐cleavage velocity per target molecule. For diagnostic purposes, the accelerated onset of the signal is more valuable than absolute peak height, as it potentially allows for shorter incubation times. Furthermore, the requirement for dual‐site recognition also imposes higher stringency (Ghouneimy et al. 2023; Morales‐Moreno et al. 2023), reducing the likelihood of off‐target activation by closely related Mycoplasma species.

The assay demonstrated 100% concordance with reference qPCR across 110 clinical samples and showed no cross‐reactivity with non‐target Mycoplasma species, including M. canis, M. felis, and bovine clinical samples positive for non‐bovis Mycoplasma spp. Importantly, all RPA‐CRISPR/Cas12a–positive samples from the genus‐level Mycoplasma group were subsequently confirmed as *M. bovis* by species‐specific qPCR, supporting assay specificity in a clinically relevant sample panel. The inverse correlation between fluorescence intensity and qPCR Ct values suggests that, while primarily qualitative, this platform retains some semi‐quantitative potential that could potentially be calibrated for bacterial load estimation in field settings.

Clinical specimens in this study included post‐mortem lung tissue, milk, nasal swabs, and nasopharyngeal swabs. However, all RPA‐CRISPR/Cas12a testing was performed using purified DNA extracts prepared through the same diagnostic workflow used for reference PCR/qPCR. Therefore, specimen matrix did not confound the comparison between assays because both methods were applied to identical purified templates. Consequently, the tolerance of the RPA‐CRISPR/Cas12a assay to matrix‐derived inhibitors or minimally processed antemortem samples was not directly evaluated and will require further validation. Lung tissue provides the highest bacterial load but restricts testing to necropsy scenarios, which can be challenging for herd health management. Future validation should focus on non‐invasive sampling methods, such as deep nasopharyngeal swabs or transtracheal washes, which are practical for live animal restraint in a chute (Schwartz et al. 2024). However, these matrices introduce new challenges, including the presence of PCR inhibitors (e.g. found in blood, mucin) and lower bacterial loads compared to consolidated lung tissue (Schrader et al. 2012). Given that RPA is generally more robust to inhibitors than PCR (Daher et al. 2016; Tan et al. 2022), this platform is well‐positioned for such samples, but analytical sensitivity must be re‐evaluated to ensure the limit of detection remains sufficient for identifying asymptomatic carriers or early‐stage infection in the upper respiratory tract. To fully realize the potential of this assay as a “pen‐side” tool, the detection modality must transition from fluorescence, which requires a fluorometer or blue‐light transilluminator, to a visual lateral flow assay (LFA). This adaptation would involve modifying the reporter topology by replacing the fluorophore‐quencher ssDNA reporter with a biotin‐FAM labeled reporter (Broughton et al. 2020). In an intact state, the reporter is captured at the control line; upon Cas12a activation and cleavage, the FAM molecule is released to bind anti‐FAM antibodies at the test line, producing a visible band (Hasan et al. 2025). While LFA formats typically sacrifice the semi‐quantitative resolution offered by fluorescence kinetics (as evidenced by our correlation with Ct values), a binary readout is preferable for field conditions where immediate, actionable decisions are required.

Despite these promising results, several limitations must be addressed before commercial or field deployment. First, the current workflow relies on a “two‐step” transfer (RPA product transferred to Cas12a mix), which introduces a risk of amplicon contamination in high‐throughput environments (Chen et al. 2022; Ghouneimy et al. 2023). Developing a “one‐pot” formulation, where Cas12a and RPA reagents coexist, remains a challenge due to the competing buffer and temperature requirements of the enzymes (Cheng et al. 2025). Second, while the assay eliminates the need for thermocyclers, it still requires DNA extraction and cold‐chain storage for the protein‐based reagents (Cheng et al. 2025; Ghouneimy et al. 2023). Finally, although the guide RNAs were designed to be sequence‐specific for *M. bovis*, the assay was not challenged against non‐mycoplasmal agents associated with bovine respiratory disease complex, such as Mannheimia haemolytica, Pasteurella multocida, and Histophilus somni, which may co‐occur with *M. bovis* in clinical specimens. Future iterations should focus on integrating rapid, instrument‐free sample preparation methods lyophilized reagents, and lateral‐flow readout formats to create a true point‐of‐care solution.

In conclusion, we have engineered and validated a highly sensitive, isothermal diagnostic platform for *M bovis* by synergizing RPA amplification with CRISPR‐Cas12a detection. This assay overcomes the intrinsic sensitivity limitations of direct Cas12a interrogation, achieving an analytical limit of detection comparable to gold‐standard qPCR (~8 copies) while eliminating the requirement for thermocycling infrastructure. The successful differentiation of *M. bovis* from closely related species confirms the assay's high specificity and utility for molecular surveillance in livestock. By establishing a robust detection method that operates at constant temperature, this study lays the foundational framework for developing rapid, instrument‐free, on‐site, lateral flow diagnostics, ultimately facilitating early intervention and improved herd health management in resource‐limited agricultural settings.

## Author Contributions

**Diba Yaghoubi:** writing – original draft, data curation, formal analysis, validation, investigation, visualization, methodology. **Viju V. Pillai:** conceptualization, project administration, funding acquisition, visualization, validation, supervision, resources, writing – review and editing, methodology.

## Ethics Statement

The authors have nothing to report.

## Conflicts of Interest

The authors declare no conflicts of interest.

## Data Availability

The data that support the findings of this study are available in the supplementary material of this article.
